# Fluid-Derived Organoids from Pleural Effusion and Ascites: Emerging Models for Drug Resistance and Personalized Oncology

**DOI:** 10.7150/jca.127511

**Published:** 2026-03-04

**Authors:** Yongyang Chen, Xiaoqing Xu, Jialin Chen, Miao Yin, Jinhui Chen, Zhanghua Qi, Ming Shi, Wenmei Su

**Affiliations:** 1 Department of Pulmonary Oncology, Affiliated Hospital of Guangdong Medical University, Zhanjiang, 524001, China.; 2 Guangdong Medical University, Dongguan, 523000, China.; 3 Dalian Medical University, Dalian, 116044, China.; 4 Guangdong Provincial Key Laboratory of Autophagy and Major Chronic Non-Communicable Diseases, Affiliated Hospital of Guangdong Medical University, Zhanjiang, 524001, China.; 5 Zhanjiang Key Laboratory of Tumor Microenvironment and Organoid Research, Zhanjiang, 524001, China.; 6 Guangdong Provincial Key Laboratory of Medical Immunology and Molecular Diagnostics, Guangdong Medical University, Dongguan, 523000, China.; 7 Guangdong Medical University, Zhanjiang, 524001, China.

**Keywords:** fluid-derived organoids, pleural effusion, ascites, drug resistance, personalized oncology

## Abstract

Malignant pleural effusion (MPE) and malignant ascites (MA) are common complications in advanced-stage cancers, often signifying disease progression and resistance to treatment. Compared to tissue biopsies or surgical specimens, materials derived from effusions offer advantages such as minimal invasiveness, ease of accessibility, and the feasibility of repeated collection during therapeutic interventions. Organoids generated from tumor cells in effusions, termed fluid-derived organoids (FDOs), have demonstrated the ability to maintain genetic heterogeneity and accurately replicate patient-specific tumor phenotypes. These characteristics position FDOs as promising models for investigating drug resistance mechanisms and informing personalized oncology strategies. In the context of lung cancer, organoids derived from pleural effusions have been employed to study acquired resistance to epidermal growth factor receptor (EGFR) tyrosine kinase inhibitors and immunotherapy. Similarly, in ovarian and gastrointestinal cancers, organoids derived from ascites have proven to be valuable platforms for examining chemotherapy resistance and conducting drug sensitivity testing. FDOs have shown significant potential for translational applications by effectively correlating *ex vivo* drug responses with clinical outcomes, thus facilitating real-time monitoring of resistance evolution. However, several challenges remain, such as achieving culture standardization, maintaining the integrity of tumor microenvironment components, and integrating with multi-omics approaches. This review provides a comprehensive overview of recent advancements in the use of pleural effusion- and ascites-derived organoids for drug resistance research, underscores their applications in personalized oncology, and explores future research directions.

## Introduction

Lung, ovarian, gastrointestinal, and pancreatic cancers continue to rank among the leading causes of cancer-related mortality globally 1. A significant proportion of patients with advanced-stage disease present with malignant effusions, most frequently malignant pleural effusion (MPE) and malignant ascites (MA) 2, 3. These effusions not only indicate disease progression but also reflect the onset of therapeutic resistance, presenting a substantial challenge in the field of oncology 2. Clinically, the presence of malignant effusions is often correlated with a poor prognosis, limited therapeutic options, and diminished quality of life 4. Therefore, the development of innovative strategies to investigate drug resistance and inform personalized therapy in this patient population is of critical importance.

Conventional preclinical models, such as two-dimensional (2D) cell lines, patient-derived xenografts (PDXs), and organoids derived from surgical or biopsy tissues, have significantly contributed to our understanding of cancer biology. Nonetheless, these models are insufficient in capturing the complexity of resistance mechanisms in advanced stages of the disease (Table 1) 5. It has been shown that 2D cultures often fail to maintain intratumoral heterogeneity and lack the structural architecture of the native tumor microenvironment 6. PDX models provide a more precise representation of tumor evolution; however, they are resource-intensive, time-consuming, and limited by the replacement of human stroma with murine stroma 7, 8. Organoids derived from surgical tissues, despite their enhanced physiological relevance, depend on specimens obtained at the time of diagnosis or surgical intervention. As a result, these organoids may not accurately represent the development of resistance mechanisms that occur during treatment 9.

In contrast, malignant effusions provide an abundant and minimally invasive source of tumor cells 10. Repeated thoracentesis and paracentesis procedures can be performed throughout the progression of the disease, allowing for real-time access to tumor material as resistance develops 11. Organoids derived from effusion-derived tumor cells, collectively known as fluid-derived organoids (FDOs), constitute a promising model system 12. The tumor cells present in malignant effusions are clones that have adapted to survive in the metastatic fluid environment. They often possess enhanced metastatic capacity, stem-like properties, and resistance to anoikis—a critical mechanism of cell death evasion linked to therapy resistance 13, 14. Consequently, FDOs may be inherently predisposed to model drug resistance at a more advanced, metastatic disease stage. Furthermore, a malignant effusion is not merely a tumor cell suspension but a complex microenvironment. It is rich in immune cells, mesothelial cells, and fibroblasts 13. Effusions frequently contain dysfunctional or suppressive immune populations, such as exhausted T cells and M2 macrophages 15. If retained or reconstituted in co-culture, these cells could make FDOs excellent models for evaluating response or resistance to immune checkpoint inhibitors. Conversely, their loss during culture might lead to an overestimation of immunotherapy efficacy. Mesothelial cells and other cavity-resident cells interact with metastatic tumor cells, providing vital survival signals and forming a key component of the metastatic niche 14. By secreting various protective factors, these cells can directly influence tumor cell sensitivity to chemotherapy or target agents. Thus, the distinct advantage of FDOs lies in their inherent complexity and context. They directly capture the cellular ecosystems of advanced metastatic sites, making them potentially more relevant than PDOs from primary tumors for modeling stage-specific drug resistance mechanisms, including metastatic adaptation, immune evasion, and microenvironment-mediated protection. In the realm of non-small cell lung cancer (NSCLC), the development of pleural effusion-derived organoids (PE-organoids) have become a focal point of research. These organoids have been instrumental in studies aimed at understanding the mechanisms underlying acquired resistance to epidermal growth factor receptor tyrosine kinase inhibitors (EGFR-TKIs). Investigations include the analysis of secondary mutations, such as T790M and C797S, as well as the exploration of bypass signaling pathways involving MET and HER2 amplification 16, 17. Similarly, in the study of ovarian cancer, ascites-derived organoids (AS-organoids) have been extensively employed to elucidate the mechanisms of resistance to platinum and taxane therapies. Additionally, these organoids have been used to predict responsiveness to targeted therapies, such as PARP inhibitors 18. In gastrointestinal and pancreatic cancers, AS-organoids have been utilized to evaluate chemotherapy efficacy and to explore personalized drug screening strategies 19.

Beyond the realm of mechanistic studies, the translational applications of FDOs are gaining increasing recognition 20. Numerous studies have demonstrated a significant concordance between the *ex vivo* drug responses of FDOs and clinical outcomes in corresponding patients, indicating that organoid-based assays may serve as predictive biomarkers for treatment efficacy 21, 22. Their ability to model clonal evolution longitudinally is particularly valuable for understanding resistance dynamics 23. However, the field is still in its early stages of development. Technical challenges associated with this approach include variable success rates across different tumor types, a lack of standardized protocols, and limited preservation of tumor microenvironment components, such as immune and stromal cells 24, 25.

The subsequent discourse will concentrate on the establishment and characteristics of FDOs, followed by an overview of the applications of pleural effusion- and ascites-derived organoids in resistance research across various cancer types. Finally, we will underscore the translational applications, current challenges, and future perspectives associated with integrating FDOs into clinical practice.

## Establishment and Features of Fluid-Derived Organoids

### Sample Collection and Cell Isolation

Effusion sample collection is generally conducted through thoracentesis or paracentesis under sterile conditions 26. Compared to surgical resections, these procedures are less invasive, allow for repeated application, and yield adequate material even from patients with poor performance status 27. The initial processing of these samples involves centrifugal separation to concentrate the cells, followed by red blood cell lysis and filtration to eliminate extraneous debris 28. Numerous studies recommend the use of density gradient centrifugation (e.g., Ficoll) to enrich tumor cells and reduce contamination by immune or mesothelial cells 29. In certain cases, fluorescence-activated cell sorting (FACS) or magnetic bead-based selection may be utilized to further enrich epithelial tumor cells. However, it is important to note that these methods may increase costs and complexity 30.

### Organoid Culture Systems

**Figure 1:**
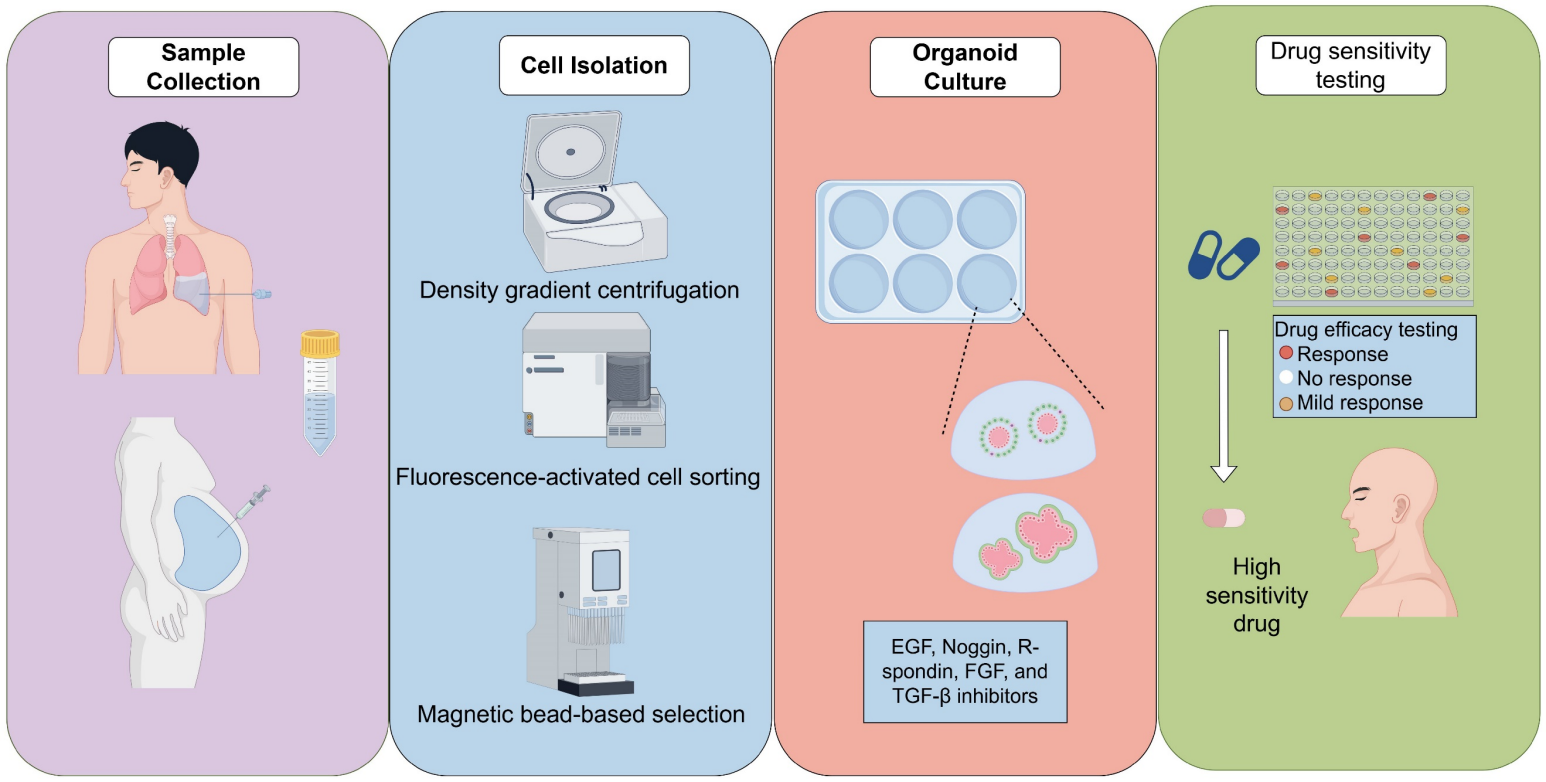
Schematic Diagram of Fluid-Derived Organoid Preparation

Isolated cells are incorporated into extracellular matrix substitutes such as Matrigel, Cultrex, or other basement membrane extracts, which provide the essential three-dimensional scaffold 31. Matrigel is a popular matrix for organoid culture but is unsuitable for clinical use due to its mouse sarcoma origin. Researchers have developed collagen-based matrices that support organoid growth like Matrigel and are more clinically viable 32. Additionally, synthetic peptide hydrogels are used for kidney organoids, improving maturation and reducing unwanted cell differentiation 33. Subsequently, these cells are cultured in optimized media formulations enriched with niche-specific growth factors and inhibitors. For lung cancer, the most prevalent media components include EGF, Noggin, R-spondin, FGF, and TGF-β inhibitors 34, 35. In the context of organoids derived from ovarian cancer ascites, the addition of Wnt agonists and estrogen-related factors has been shown to enhance growth promotion 36. The variability in media formulations across different laboratories presents a significant challenge, as it affects organoid morphology, growth efficiency, and drug response profiles (Fig. 1).

### Success Rates Across Tumor Types

As evidenced by existing literature, the efficiency of FDO establishment exhibits significant variability across different tumor types 37. The reported success rates are notably higher in ovarian cancer, ranging from 60% to 90%, attributed to the high concentration of viable tumor cells present in ascites 38. Conversely, success rates for FDO establishment from pleural effusions in lung cancer range between 30% and 60%, influenced by factors such as tumor cell fraction and prior treatment exposure 39, 40. Gastric and pancreatic cancers exhibit intermediate success rates, approximately 40% to 70%, whereas breast cancer and lymphoma demonstrate lower efficiencies 41. Key factors impacting these success rates include tumor cellularity, the extent of immune cell infiltration, and previous chemotherapy exposure 42. These findings indicate that while FDO generation is achievable across various cancer types, further optimization is necessary to ensure consistency 43.

### Preservation of Tumor Characteristics

Several studies have corroborated that FDOs maintain critical histopathological and molecular characteristics of the original tumors 44. Histological examination using hematoxylin and eosin staining frequently reveals glandular or papillary structures akin to those of the primary tumor 45. Genomic analyses further confirm that FDOs retain essential driver mutations, copy number alterations, and mutational signatures present in the original tumors in patient samples 46. Intra-patient heterogeneity is notably preserved, as organoids can reflect subclonal populations identified in sequencing studies 47. Further evidence of their fidelity is demonstrated through transcriptomic profiling, which indicates that FDOs cluster closely with their corresponding parental tumors 48.

### Characteristics of FDOs

Compared to tissue-derived patient-derived organoids (PDOs), FDOs present several advantages (Table 2). Firstly, they are minimally invasive and can be repeatedly sampled, facilitating longitudinal studies 49. Secondly, effusions are often enriched with metastatic tumor cells, potentially offering a more accurate representation of advanced disease biology than resected primary tumors 50. Lastly, the establishment of FDOs does not require surgical intervention, thereby broadening their applicability to patients with inoperable or late-stage disease 51.

Despite the advantages, several challenges remain. Effusion samples are inherently heterogeneous, often comprising immune cells, fibroblasts, and mesothelial cells 52. Although a certain level of heterogeneity is generally deemed beneficial, it is crucial to recognize that an overabundance of non-tumor cells can impede the establishment of organoids 53. Long-term culture tends to select for epithelial cells, leading to the depletion of stromal and immune components that are essential for accurately modeling tumor-microenvironment interactions 54. Additionally, the lack of standardized protocols across laboratories hampers reproducibility and complicates cross-study comparisons 55. Scalability also presents a challenge: while individual FDO cultures can be expanded for drug testing, the creation of large biobanks for high-throughput screening demands substantial resources 56.

FDOs occupy a distinct position within the spectrum of preclinical models. While 2D cell lines are well-documented for their cost-effectiveness and scalability, they fall short in representing cellular heterogeneity 57. PDX models, on the other hand, maintain tumor complexity but are hindered by lengthy establishment times and the confounding presence of murine stroma 58. Although tissue-derived PDOs offer physiological relevance, their utility is constrained by challenges related to surgical access and the initial sampling process. In contrast, FDOs provide a unique combination of accessibility, fidelity to the original tumor, and the capacity for longitudinal sampling, thereby serving as a complementary asset in the array of preclinical models 59.

## Pleural Effusion-Derived Organoids in Drug Resistance Research

MPE is a common complication associated with advanced-stage malignancies, with lung cancer being the most frequent underlying cause 60. Approximately 40% of patients with advanced NSCLC experience pleural effusions during the course of their illness, particularly in the context of treatment resistance and disease progression 61. This study highlights the potential of generating organoids from pleural effusion-derived tumor cells (PE-organoids), providing a unique platform to explore resistance mechanisms, monitor disease evolution over time, and assess personalized therapeutic strategies 62.

### Lung Cancer: EGFR-TKI Resistance

NSCLC, especially the adenocarcinoma subtype, is strongly linked to activating mutations in the EGFR 63. Although EGFR tyrosine kinase inhibitors (EGFR-TKIs) have significantly improved outcomes for patients with EGFR-mutant NSCLC, it is crucial to recognize that nearly all patients eventually develop acquired resistance to these therapies 64. To investigate this phenomenon in greater detail, research has utilized patient-derived *ex vivo* PE-organoids obtained from pleural effusion samples. These models have been employed to elucidate resistance mechanisms 65. The secondary EGFR T790M mutation is responsible for approximately 50% of resistance to first- and second-generation TKIs and has been accurately maintained in PE-organoids, facilitating the functional assessment of third-generation TKIs such as Osimertinib 66. Prolonged treatment with osimertinib leads to the emergence of additional resistance mechanisms, including the EGFR C797S mutation and activation of bypass pathways such as MET or HER2 amplification and alterations in the PI3K pathway 67, 68. The modeling of these resistance features in PE-organoids has been a productive endeavor, as demonstrated by the successful documentation of the molecular evolution of resistance 69. Beyond EGFR, PE-organoids have also been utilized to explore resistance in ALK-rearranged NSCLC 70. Organoids derived from EML4-ALK-positive effusions have revealed secondary ALK mutations (e.g., L1196M, G1202R) that confer resistance to second-generation ALK inhibitors 71. These findings underscore the versatility of PE-organoids as platforms for investigating resistance across various oncogene-driven subsets of NSCLC.

### Immune Resistance in NSCLC

Immunotherapy utilizing immune checkpoint inhibitors (ICIs), particularly those targeting the PD-1/PD-L1 axis, has emerged as a fundamental component in the treatment of NSCLC 72. Nevertheless, therapeutic responses are limited to a subset of patients, and the phenomenon of acquired resistance is becoming increasingly prevalent 73, 74. Although most PDO systems are devoid of immune elements, pleural effusions frequently contain tumor-associated lymphocytes and macrophages 75, 76. By co-culturing pleural effusion-derived organoids with autologous immune cells from the same effusion, researchers have begun to investigate mechanisms of immune resistance, such as alterations in macrophage polarization that may contribute to sustained resistance to PD-1 blockade 77. These co-culture models hold promise for evaluating combination therapies designed to overcome immune resistance.

### Other Cancers with Malignant Pleural Effusion

While lung cancer is the predominant cause of MPE, such effusions also occur in breast cancer, gastric cancer, and hematologic malignancies 78, 79. Although research on breast cancer pleural effusion-derived organoids is limited, organoid models have been effectively employed to study resistance to endocrine and HER2-targeted therapies in breast cancer, suggesting that effusion-derived organoids could be similarly utilized for these investigations 80. In gastric cancer, PE-organoids have been developed to assess sensitivity to platinum-based chemotherapy, yielding results that align with patient responses and thereby underscoring their predictive utility 81-83. Initial efforts to culture organoids from lymphoma-associated pleural fluid have also been documented 84.

### Longitudinal Monitoring of Resistance Evolution

A distinct advantage of PE-organoids is their applicability for longitudinal monitoring 85. Patients experiencing recurrent MPE can undergo multiple thoracenteses, providing sequential samples that capture tumor evolution under therapeutic pressure 86. Serial PE-organoids have effectively documented the transition from EGFR exon 19 deletion-sensitive disease to T790M-positive resistance, and subsequently to C797S-mediated resistance 87. Drug testing on these sequential organoids has mirrored clinical outcomes, demonstrating the feasibility of real-time resistance monitoring 88. Longitudinal sampling not only corroborates clinical observations but also reveals novel resistance mechanisms 89. Changes such as the activation of epithelial-mesenchymal transition (EMT), histological transformation from adenocarcinoma to small cell lung cancer, and modifications in the tumor microenvironment have been identified in serial PE-organoids, underscoring the value of effusion-derived models for studying tumor plasticity and heterogeneity 90.

### Advantages and Limitations of PE-Organoids

PE-organoids present several advantages, including minimally invasive sampling, the capacity to investigate advanced stages of disease, and a high degree of concordance with clinical resistance phenotypes 80. They serve as a complement to tissue-derived PDOs and circulating tumor cell (CTC)-derived models by providing an abundant and viable source of tumor cells directly from metastatic lesions 91. However, PE-organoids are established with less efficiency than ascites-derived organoids, primarily due to the lower concentration of tumor cells present in pleural fluid 92. Additionally, over time, the immune and stromal components within the culture tend to diminish 93. To facilitate their integration into routine clinical workflows, there is a need for both standardized culture methods and extensive clinical validation (Table 3).

## Ascites-Derived Organoids in Drug Resistance Research

MA is commonly observed in individuals with advanced stages of ovarian, gastric, pancreatic, and other abdominal cancers 94, 95. The ascitic fluid is a rich reservoir of exfoliated tumor cells, immune cells, and stromal components, rendering it particularly advantageous for the cultivation of organoids 96. In comparison to pleural effusions, malignant ascites typically exhibits higher tumor cellularity, which contributes to increased success rates in the establishment of organoids 97. Organoids derived from ascites (AS-organoids) have been extensively utilized to model chemotherapy resistance, evaluate sensitivity to targeted therapies, and investigate personalized treatment strategies 18.

### Ovarian Cancer

Among various cancers, ovarian cancer exhibits the strongest association with malignant ascites 98. Although platinum- and taxane-based regimens remain the standard therapeutic approach, the majority of patients experience relapse due to the development of acquired resistance 99. AS-organoids derived from ovarian cancer ascites effectively replicate the phenotypes associated with chemotherapy resistance and maintain critical genomic alterations pertinent to therapeutic interventions 18. Furthermore, they facilitate the assessment of sensitivity to targeted therapies, such as PARP inhibitors, thus serving as *ex vivo* platforms for predicting treatment response 100, 101.

### Gastrointestinal and Pancreatic Cancers

In gastrointestinal malignancies, such as metastatic colorectal cancer, organoids derived from ascitic fluid have been employed for drug sensitivity testing of fluoropyrimidines and platinum-based agents, demonstrating a correlation between organoid responses and patient outcomes 102. Additionally, ascites-derived organoids have been utilized to explore resistance mechanisms to HER2-targeted therapies in HER2-positive gastric cancer 103. In the realm of pancreatic cancer, organoids derived from ascites have been instrumental in drug screening efforts, including the assessment of FOLFIRINOX and gemcitabine/nab-paclitaxel treatment regimens, although research in this area remains limited 104-106.

### Longitudinal Monitoring and Tumor Evolution

The recurrent nature of ascites allows for repeated sampling, facilitating the establishment of sequential organoids from the same patient 92. This methodology has been applied to track the evolution of resistance in ovarian cancer, capturing transitions from platinum-sensitive to platinum-resistant states and identifying secondary BRCA mutations that restore homologous recombination 107, 108. Furthermore, longitudinal monitoring of patient-derived pancreatic ductal adenocarcinoma (PDAC) organoids, utilizing live-cell imaging and deep learning techniques, has enabled dynamic evaluation of treatment responses, thereby providing a framework for capturing tumor evolution under therapeutic pressure 109.

### Advantages and Limitations of AS-Organoids

AS-organoids typically demonstrate higher establishment rates and extended culture viability compared to PE-organoids, primarily due to the increased tumor cellularity present in ascites samples 110. This characteristic is particularly advantageous in the context of ovarian cancer, where malignant cells are abundant. Additionally, the larger volumes of these samples facilitate higher-throughput drug screening 111. However, it is important to note that stromal and immune components are gradually lost during prolonged culture periods, and reactive mesothelial cells may occasionally proliferate more rapidly than tumor cells, thereby complicating the culture process 112. Furthermore, organoids derived from gastrointestinal and pancreatic cancers remain relatively underexplored, necessitating further validation of their clinical relevance. AS-organoids serve as *ex vivo* platforms for evaluating chemotherapies, targeted agents, and novel drug combinations, with the potential to personalize therapeutic approaches for patients with recurrent ascites 113. The integration of genomic and transcriptomic analyses can enhance patient stratification and facilitate the identification of resistance biomarkers 114. Ultimately, AS-organoids hold the potential to be incorporated into prospective clinical trials as predictive assays, thereby guiding individualized treatment strategies (Table 3) 115.

### Resistance Mechanisms Across Cancer Types

Despite originating from different anatomical sites (pleural cavity vs. peritoneal cavity) and diverse cancer types, the acquired resistance mechanisms revealed by FDOs demonstrate remarkable commonalities. These shared mechanisms transcend specific histological origins, reflecting universal adaptive strategies of tumors under therapeutic pressure. The activation of EMT has been identified in longitudinal FDO models of both lung and ovarian cancers, associating with a more aggressive and therapy-resistant phenotype. Secondary driver gene mutations (e.g., EGFR T790M/C797S and ALK L1196M in lung cancer; BRCA reversion mutations in ovarian cancer) represent a key pathway for evading targeted therapies. The activation of bypass signaling pathways (e.g., MET/HER2 amplification, PI3K pathway alterations) is a common strategy for resistance to targeted agents across multiple cancers. Furthermore, alterations in the tumor microenvironment, including immune cell dysfunction (e.g., T-cell exhaustion, M2 macrophage polarization) and protective signals from stromal cells (e.g., mesothelial cells), collectively constitute a niche that supports tumor survival and mediates resistance to chemotherapy, targeted therapy, and immunotherapy. Highlighting these commonalities underscores the value of FDOs as powerful tools for systematically studying the universal principles of resistance biology across cancer types (Table 4).

## Translational Applications of FDOs in Personalized Oncology

### *Ex vivo* Drug Sensitivity Testing

*Ex vivo* drug screening using organoids represents a promising approach for guiding therapeutic interventions 65. are particularly advantageous due to the ease of obtaining effusion samples, even from patients with advanced-stage disease 116. By cultivating tumor cells from pleural effusion or ascitic fluid within three-dimensional systems, FDOs can be evaluated against various therapeutic agents, with several studies demonstrating a correlation between these responses and patient outcomes 117. The ability to repeatedly sample effusions facilitate the generation of sequential organoids from the same patient throughout the course of treatment. This capability allows for the dynamic investigation of resistance evolution and treatment adaptation, as serial pleural effusion- and ascites-derived organoids reflect clinical progression 118. Sequencing FDOs offers insights into the molecular drivers of resistance and supports patient stratification for targeted therapies 119. Furthermore, transcriptomic and single-cell analyses elucidate pathway reprogramming, immune evasion, and metabolic alterations, which can guide the development of rational therapeutic combinations 120. Effusion samples frequently contain immune cells, enabling the establishment of co-culture systems with autologous lymphocytes or macrophages to explore mechanisms of immune resistance 121. These models may improve prediction of ICI efficacy and support testing of combinations with targeted agents or PARP inhibitors 122.

### Biobanking and Clinical Trials

Biobanks of FDO derived from pleural effusion and ascites samples serve as valuable resources for translational research and precision medicine trials 123. Emerging prospective studies are integrating organoid-based assays into clinical decision-making processes, thereby establishing a foundation for large-scale validation 124. Prior to widespread implementation, several challenges must be addressed, including the standardization of protocols, optimization of assay turnaround times, development of regulatory frameworks, and assessment of cost-effectiveness. Adequately powered prospective trials are necessary to substantiate that FDO-guided treatments enhance patient outcomes compared to standard care.

## Technical and Standardization Challenges

The absence of standardized protocols for the establishment of FDOs leads to significant variability across laboratories in terms of sample processing, matrix selection, and media composition. The success rates of these protocols are contingent upon the type of tumor, which is further influenced by factors such as tumor cellularity, immune infiltration, previous treatments, and handling conditions 92, 125. For the advancement of multi-center studies, it is imperative to develop reproducible and cost-effective protocols 126. The immune, stromal, and mesothelial components present in effusions are frequently lost during culture, which restricts the ability to model interactions between tumors and their microenvironment 127. To address this limitation, co-culture strategies involving autologous immune cells or cancer-associated fibroblasts, as well as organoid-on-chip platforms, are being developed to more accurately replicate the native microenvironment 128. The integration of functional organoid data with genomic, transcriptomic, proteomic, and metabolomic analyses presents a complex challenge that necessitates robust bioinformatic infrastructure. Although artificial intelligence and machine learning approaches have the potential to synthesize high-dimensional data to predict drug responses, they require extensive validation 129, 130. The expansion from single-patient cultures to large-scale biobanks demands coordinated infrastructure, standardized protocols, and adequate resources 131. Comprehensive repositories of PE and AS organoids will significantly enhance drug discovery and preclinical validation 132.

The translation of FDO-based functional diagnostic tests from research tools to clinically actionable assays face significant regulatory hurdles, primarily centered on CLIA (Clinical Laboratory Improvement Amendments) certification and analytical validation 133. For an FDO drug sensitivity test to be used in guiding patient therapy in the United States, it must be performed in a CLIA-certified laboratory 133, 134. This mandates the development of and adherence to stringent, standardized protocols for every step-from sample processing and organoid culture to drug exposure and viability readout-ensuring rigorous quality control, personnel competency, and assay reproducibility 133.

The current lack of standardized protocols for FDO generation poses a fundamental challenge to meeting these requirements. Beyond laboratory standards, the assay itself must undergo extensive analytical validation to demonstrate its reliability. This involves formally establishing key parameters such as precision, accuracy, sensitivity, sample stability, and assay robustness 135. Ultimately, establishing clinical validity-proving that the test result predicts patient outcome through large-scale prospective trials-is the critical final step. The path to regulatory approval thus requires a coordinated process of protocol standardization, analytical validation within a CLIA framework, and subsequent clinical validation, which represents a substantial but necessary undertaking for integrating FDO-based functional diagnostics into routine clinical practice.

Substantial evidence from prospective trials is essential to confirm the efficacy of FDO-guided treatment 136. Critical considerations encompass turnaround time, assay thresholds for sensitivity and resistance, and the integration of findings into tumor boards 137. The direct application of FDOs to guide clinical therapy selection or monitor resistance remains predominantly at the preclinical validation or early-stage clinical research phase, while the field is promising, studies providing definitive clinical predictive data are still limited. A clinical study specifically designed for FDOs, illustrating the complete pathway from research objectives to clinical application validation (NCT06658080), To evaluate the accuracy of FDOs in predicting the efficacy of chemotherapy/targeted therapy in patients with advanced breast cancer. The treatment decision window for advanced cancer is typically short (approximately 2-4 weeks). While traditional organoid culture requires 4-8 weeks, integrating new technologies like microfluidics has the potential to shorten the entire workflow to 1-2 weeks, which is crucial for real-time treatment guidance. Not all effusion samples can successfully yield FDOs, as this depends on the viability and quantity of tumor cells in the sample. Furthermore, the ability to retain key tumor microenvironment components during culture directly impacts the predictive accuracy for therapies like immunotherapy. Currently, there is a lack of uniform standards for FDO culture, drug testing, and result interpretation, making data comparison across different laboratories difficult. Additionally, the high cost of complex 3D culture and personalized testing presents a barrier to widespread adoption. Most current supporting evidence comes from retrospective or small-scale prospective studies. To establish the clinical utility of FDOs, it is essential to demonstrate through large-scale, prospective, interventional clinical trials that their use ultimately improves patient survival or quality of life. In summary, FDOs represent a highly promising tool for achieving precision cancer therapy, particularly in modeling late-stage metastatic foci and guiding treatment for refractory patients. However, their clinical application still faces practical challenges such as long turnaround times and a lack of standardization. This field is in a period of rapid development, and the integration of new technologies is key to overcoming existing bottlenecks.

Regulatory frameworks must address issues of reproducibility, quality control, and patient safety 138. Promising avenues for advancement include the development of organoid-on-chip systems, immune-integrated organoids, AI-driven analytics, global biobanking networks, and the incorporation of these technologies into clinical trials. The convergence of technological innovations with rigorous clinical validation has the potential to transform FDOs from experimental tools into vital components of precision oncology.

## Conclusion

Organoids derived from malignant effusions-pleural fluid and ascites-have established a powerful and clinically accessible platform for studying drug resistance. These FDOs complement existing models by capturing dynamic tumor evolution through serial sampling and preserving patient-specific resistance profiles. Although challenges such as variable culture success, protocol standardization, and loss of native microenvironment components remain, ongoing technological advances-in co-culture systems, organoid-on-chip platforms, and multi-omics integration-are actively addressing these gaps. Moving forward, establishing well-characterized FDO biobanks and incorporating organoid-guided assays into prospective clinical trials will be essential to validate their predictive utility. In summary, FDOs offer a minimally invasive, physiologically relevant, and highly adaptable model system that holds strong potential to illuminate resistance mechanisms and inform personalized treatment strategies in advanced cancer.

## Figures and Tables

**Table 1:** Comparison of FDOs with other preclinical models

	Advantage	Disadvantage	References
2D cell lines	Low cost, high throughput	Lack of heterogeneity and three-dimensional structure	6
PDXs	Preserve tumor complexity	Mouse-derived matrix interference, time-consuming	7,8
Organoids from surgical tissues	High physiological relevance	Relying on surgical samples, unable to monitor dynamically	9
FDOs	Minimally invasive, repeatable sampling, representative of advanced disease	The success rate varies greatly depending on the type of tumor	12-14
Co-culture system	Simulated immune microenvironment	Technically complex and low in standardization	120,127

**Table 2:** Establishment and Characterization Comparison of Malignant Pleural and Ascitic FDOs

	Sample Collection	Cell Isolation and Enrichment	Key components in Culture Medium	Drug Resistance Mechanism Simulation	Sampling Feasibility
**PE-organoids**	Thoracentesis	Density gradient centrifugation	EGF, FGF, TGF-β inhibitor	EGFR-TKI resistance mutations (T790M)	High (repeatable puncture)
**AS-organoids**	Paracentesis	Magnetic bead separation/FACS	Wnt agonist, estrogen factor	Platinum/Taxane Resistance	High (common in recurrent ascites)
**PDOs**	Surgical biopsy	Mechanical/enzymatic digestion method	Tissue-specific cytokines	Primary tumor drug resistance profile	Low (surgery-dependent)

**Table 3:** Application of PE-organoids and AS-organoids

Application of PE-organoids in drug resistance research					
	**Main drug resistance mechanisms**	**Drug test results and clinical relevance**	**Microenvironment retention status**	**Culture success rate**	**References**
**NSCLC**	1.EGFR T790M mutation 2. MET/HER2 amplification 3.PI3K pathway activation	1. The prediction accuracy for osimertinib sensitivity is 85%. 2. The response rate to immunotherapy is related to PD-L1 expression.	Partial retention of lymphocytes and macrophages	30%-60%	15,16,62,69
**Breast cancer**	Abnormal HER2 signaling pathway, endocrine therapy resistance (ESR1 mutation)	HER2-targeted drug response is consistent with patient clinical	Significant loss of stromal cells	20%-40%	77-79
**Lymphoma**	1.B cell receptor signaling pathway activation 2.Immune checkpoint inhibitor resistance	The results of chemotherapy drug screening are related to patient survival	Lack of B cell-T cell interaction	<20%	40,48,83
**Gastric Cancer**	1.Loss of HER2 amplification 2. FGFR2 overexpression	Trastuzumab resistance is consistent with HER2 downregulation in organoids.	Co-culturing with fibroblasts can enhance model stability.	40%-50%	77,78,102
**Application of AS-organoids in drug resistance research**					
**Ovarian cancer**	1.BRCA reversion mutation 2.Homologous recombination repair restoration 3.Upregulation of platinum efflux pumps	PARP inhibitor sensitivity prediction accuracy is 80%	Preserve CA125 secretion function	60%-90%	17,35,37
**Colorectal cancer**	1.KRAS/BRAF mutations 2.EGFR monoclonal antibody resistance (MET amplification)	Cetuximab resistance is consistent with KRAS mutations in organoids.	Co-culture tumor-associated macrophages	50%-70%	101,111
**Pancreatic cancer**	1.Stroma-mediated chemotherapy barrier 2.Deficiency of gemcitabine-metabolizing enzymes	FOLFIRINOX response prediction is associated with patients' progression-free survival	Stromal cells need to be added to maintain the viability of the model.	40%-60%	18,103-106

**Table 4:** Resistance mechanisms across cancer types modeled by FDOs

Resistance Mechanism	Pleural Effusion-Derived Organoids	Ascites-Derived Organoids	References
**Secondary Driver Gene Mutations**	EGFR T790M/C797S (vs. EGFR-TKIs); ALK L1196M/G1202R (vs. ALK inhibitors)	BRCA1/2 reversion mutations (restoring HR, vs. PARP inhibitors)	65-68, 70, 71, 106, 107
**Bypass Pathway Activation**	MET/HER2 amplification, PI3K pathway alterations (vs. EGFR/ALK inhibitors)	Understudied (likely involves RTK reprogramming)	66-68
**Histological / Cell Fate Transition**	Adenocarcinoma to SCLC transformation; EMT activation	EMT activation; Dedifferentiation	89, 108
**Altered DNA Damage Response**	Possibly involved (linked to SCLC transformation)	Restored homologous recombination	89, 106, 107
**Microenvironment-Mediated Protection**	Immune suppression (exhausted T cells, M2 macrophages); Survival signals from mesothelial cells	Immune suppression; Stromal cell interactions; Unique cytokine milieu of ascites	74-76, 95, 111
